# Hydrogenated Amorphous Silicon-Based Nanomaterials as Alternative Electrodes to Graphite for Lithium-Ion Batteries

**DOI:** 10.3390/nano12244400

**Published:** 2022-12-09

**Authors:** Rocío Barrio, Nieves González, Álvaro Portugal, Carmen Morant, José Javier Gandía

**Affiliations:** 1 Centro de Investigaciones Energéticas, Mediambientales y Tecnológicas, Avenida Complutense 40, CP-28040 Madrid, Spain; 2 Department of Applied Physics, Instituto de Ciencias de Materiales Nicolás Cabrera, Autonomous University of Madrid, CP-28049 Madrid, Spain

**Keywords:** anodes, lithium-ion battery, amorphous silicon, amorphous silicon nanowires, energy storage

## Abstract

Graphite is the material most used as an electrode in commercial lithium-ion batteries. On the other hand, it is a material with low energy capacity, and it is considered a raw critical material given its large volume of use. In the current energy context, we must promote the search for alternative materials based on elements that are abundant, sustainable and that have better performance for energy storage. We propose thin materials based on silicon, which has a storage capacity eleven times higher than graphite. Nevertheless, due to the high-volume expansion during lithiation, it tends to crack, limiting the life of the batteries. To solve this problem, hydrogenated amorphous silicon has been researched, in the form of thin film and nanostructures, since, due to its amorphous structure, porosity and high specific surface, it could better absorb changes in volume. These thin films were grown by plasma-enhanced chemical vapor deposition, and then the nanowires were obtained by chemical etching. The compositional variations of films deposited at different temperatures and the incorporation of dopants markedly influence the stability and longevity of batteries. With these optimized electrodes, we achieved batteries with an initial capacity of 3800 mAhg^−1^ and 82% capacity retention after 50 cycles.

## 1. Introduction

In the current context of energy transition towards a more sustainable model based on the production of energy from renewable sources, the energy storage will play a fundamental role due to the intermittent nature of this type of energy systems and the mismatch that occurs between the generation and its demand [1]. Overall, developing novel solar devices, batteries and recycling technologies requires materials-based solutions in order to prevent the climate catastrophe and the energy dependence that it portends [2].

It is currently believed that the lithium-ion battery (LIB) is one of the best technologies among the available storage systems. This largely stems from huge utilization by portable electronic devices, such as smartphones, laptops, drones, etc., which use this type of batteries, as well as the great boost that the electric vehicle has undergone in recent years and for the necessary energy storage from renewable but intermittent energy sources, such as solar and wind power. In addition, this type of battery exhibits excellent features, among which its high energy and power density, a high operating voltage and its durability stand out. The impact of this technology is so great that its development has been considered one of the greatest challenges achieved in recent decades in the scientific and technological fields. So much so that in 2019, the Nobel Prize in Chemistry was awarded to the researchers who contributed to developing this type of lithium-ion battery [3]. However, even though this technology has achieved adequate maturity and is commercially available, it still has to address great challenges such as cost reduction to enhance its feasibility (especially in large-scale renewable energy installations that need high investment) and using abundant and sustainable materials and electrolytes, as well as investigating recycling and reuse. Among the challenges mentioned above, we have focused on researching renewed materials as electrodes in LIB, in the study of hydrogenated amorphous silicon as anode (negative electrode), which is the subject of this work. 

**Figure 1:**
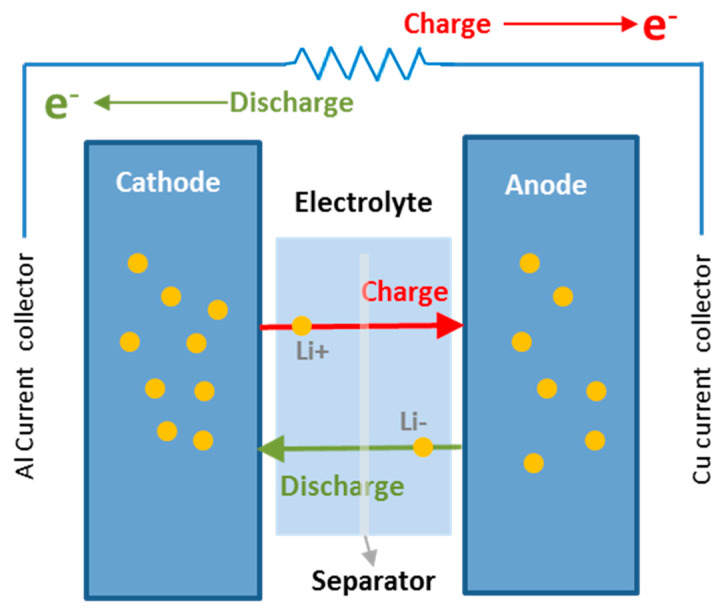
Configuration and operating principle of a lithium-ion battery.

A lithium-ion battery consists of two electrodes immersed in an electrolyte solution and separated from each other by a membrane that functions as an electrical insulator. The porous design ensures that only lithium ions can pass through the membrane and therefore move between electrodes. Furthermore, it isolates the two electrodes from each other so that internal short circuits do not occur. During the charging process, a flow of electrons moves in an external circuit from the positive electrode (cathode) to the negative electrode (anode) where lithium ions from the cathode are reduced after scrolling through the electrolyte [4,5,6]. During discharge, the process is reversed and both lithium ions and electrons move towards the cathode where the reduction of lithium occurs, as illustrated in Figure 1. The battery is fully charged when there is no flow of lithium ions through electrolyte [7]. The more lithium ions can be housed in an anode material the higher the capacity of the battery will be.

### Silicon vs. Graphite as Electrode

So far, graphite is the most widely used material as anode in commercial LIBs [4]. This carbon-based electrode has great advantages such as good electrochemical performance, high cyclability, slight volume changes during the charge and discharge processes (less than 20%) and low cost. On the contrary, the European Commission has estimated that graphite may become a moderate critical material for the development of this technology, since its growing demand for energy storage could provoke a lack of supply [8] despite the abundance of carbon in the crust of the earth. This fact of a lack of graphite for LIBs could give rise to a bottleneck in the development of the energy transition and the electric vehicle.

On the other hand, pure graphite anodes have reached their maximum performance concerning energy density. This material has low theoretical storage capacity (372 mAhg^−1^), which may be a significant limitation in some applications. Therefore, it is mandatory to find alternatives materials that are also abundant, sustainable and, as much as possible, have better electrochemical features than graphite.

In comparison, silicon is another very abundant element in the terrestrial crust and has the highest theoretical lithium storage capacity (4200 mAhg^−1^, that is, eleven times greater than graphite), and it is also not particularly expensive, which has led to the consideration of silicon as a good candidate to replace graphite as anode in the next generation of LIBs [6,7]. Its greater storage capacity could allow for lighter batteries than current LIBs. However, during lithiation and delithiation, crystalline silicon undergoes huge volume changes (higher than 300%) that deteriorate the material and ultimately cause it to fracture and detach from the metal collector, leading to failure and shortening the battery lifetime. Another drawback associated with silicon is its low electrical conductivity compared to graphite (1 S·cm^−1^), which limits rapid discharge applications [8,9] although this could be solved to some extent by introducing dopant impurities into the material (boron for p-type doping and phosphorous for n-type doping) to increase its conductivity and consequently the battery performance. 

Owing to the good characteristics provided by silicon as anode for LIBs, the scientific community and some R&D departments of the industry are currently working in growing silicon-based materials conformed into nanostructures or thin films as a strategy to mitigate the consequences from volume changes and prevent the premature failure of the batteries [10,11,12,13,14,15,16,17]. 

In this work, we have proposed intrinsic hydrogenated amorphous silicon (a-Si:H (i)) deposited in thin films (less than a micrometer) by plasma-enhanced chemical vapor deposition (PECVD) [13] as an alternative anodic material to graphite. Unlike crystalline silicon, the amorphous nature of a-Si:H and its high porosity allow it to better withstand changes in volume and promote the diffusion of lithium ions in the material. Furthermore, a-Si:H can be directly deposited on the current collector and presents good adhesion, so there is no need to use binders, which are required, for example, in the case of crystalline silicon electrodes. Its electrical conductivity can be easily improved by introducing gases with dopant elements in the starting mixture [14] to obtain n-doped hydrogenated amorphous silicon (a-Si:H(n)). In turn, a-Si:H obtained by PECVD is more economically viable than crystalline silicon. 

In order to increase the specific area of the electrode with respect to its volume and thus achieve higher capacity, we have also examined the formation of amorphous silicon nanowires (a-SiNWs) produced by subjecting a hydrogenated amorphous silicon film previously deposited on a copper foil to an etching process called MACE (metal-assisted chemical etching) [18,19,20]. With this method, binders of the silicon nanowires on the copper collector are not required, unlike other techniques for growing crystalline (non-amorphous) silicon nanowires [16,21].

After the optimization of these intrinsic and n-doped hydrogenated amorphous silicon films deposited by PECVD and nanowire formation by MACE on the intrinsic films, the electrodes made of these materials are assembled into coin cell batteries to assay their feasibility as alternative anodes to graphite. The first results obtained using these three materials have been very satisfactory, with initial discharge capacity greater than 3800 mAhg^−1^ and capacity retention higher than 82% after 50 charge and discharge cycles.

## 2. Materials and Methods

### 2.1. Preparation and Characterization of Hydrogenated Amorphous Silicon (a-Si:H)

For years, authors has intensity worked in the development and optimization of N and P doped and intrinsic hydrogenated amorphous silicon thin films by PECVD [17,19,22,23], in thin film solar cells with an amorphous slicon P-I-N structure and in silicon heterojunction solar cells (SHJ) [24,25,26,27]. Our previous experience has been very useful in this work to establish what requirements the silicon-based material must meet for its application as anode in LIBs. Our extensive experience in nanomaterials and the assembly and characterization of lithium-ion batteries [18,19,28,29,30] made possible this application. Thus, we have been able to expand the adaptability of our PECVD equipment for other fields of applications, as well as to take advantage of the multifunctionality of our own a-Si:H material in terms of energy (solar and storage).

Thin-film growth technology by PECVD offers important advantages, such as operating temperatures below 250 °C (which represents considerable energetic savings compared to other technologies) and a good homogeneity of the films, and it is possible to easily control and tunnel both the thickness and the properties of the material by acting on the parameters of the deposition process. The PECVD system employed for this study is an MVSystem trademark equipment with an excitation frequency of 13.56 MHz, which uses pure silane as precursor gas (SiH_4_, 99.999% Nippon). Occasionally, a flow of 2% phosphine gas diluted in hydrogen gas was added to the gas mixture to increase the electrical conductivity of material obtaining n-type doped hydrogenated amorphous silicon (a-Si:H(n)).

The deposited a-Si:H(i) and a-Si:H(n) films were optically and electrically characterized. For the optical characterization, transmittance and reflectance measurements of samples deposited on glass were obtained by using a U*V/V*IS/IR Lamba1050-Perkin Elmer spectrophotometer, in the range between 300 nm and 2500 nm. From these spectra, the thickness and the average refractive index (n_average_) were determined according to a procedure described in previous works [31], in order to test the quality of the material. In this work, the refractive index is the average value of the curve of refractive index in the range of spectra (i.e., from 300 to 2500 nm). The dark conductivity (σ) of the n-doped samples was measured from the current voltage characteristics (I–V) using a Keithley 617 electrometer at room temperature. It was realized in a coplanar configuration with evaporated aluminum contacts on top of the films. In addition, we analyzed the behavior of the conductivity versus temperature to determine the activation energy (E_a_), which allowed us to evaluate the doping level. 

Regarding the structure of the material, its hydrogen content and its bond arrangement were determined from the infrared absorption spectra obtained by a Perkin Elmer Lamda 100FT-IR spectrophotometer [32]. These measurements were accomplished on a-Si:H films deposited on high-resistivity monocrystalline silicon wafers. This technique provides us with information about the probable stoichiometry of the material, taking account the locations and the relative intensities of the characteristic bands in its spectrum. Thus, from the rocking and wagging bands located at 635 cm^−1^ we can determine the hydrogen content of the material. Instead, the stretching bands centered at 2000 cm^−1^ and 2100 cm^−1^ denote how the hydrogen is bonded in its structure, that is, forming monohydride bonds (Si-H, band at 2000 cm^−1^) and polyhydride bonds (Si-H_x_, band at 2100 cm^−1^) [33,34]. The analysis of the hydrogen content at 635 cm^−1^ [H_635_] and its distribution in hydrogen content as monohydrides [Si-H] and as polihydrides [Si-H_x_] was performed following the method of [35] with the corrected proportionally factors of Langford et al., at 635, 2000 and 2100 cm^−1^, respectively [33]. We have defined the factor R as the ratio between the intensities corresponding to the bands at 2100 cm^−1^ and 2000 cm^−1^ (I_2100_/(I_2000_ + I_2100_), which represents the proportion of hydrogen incorporated in the form of polyhydrides with respect to the total hydrogen in the material. This ratio R allows us to asses the quality of the material, since the Si-H_x_ bonds defined as polyhydrides are concentrated inside of microcavities and holes of the material. A high proportion of these Si-H_x_ bonds indicates a porous material with a high number of defects. 

In previous research by the authors [19], we have exhaustively studied the preparation conditions of a-Si:H that resulted in the formation of nanowires (a-SiNWs) with optimal lengths and density for their application as anodes in LIBs. From this study, we were able to deduce how small deviations in the hydrogen content and its arrangement in the material greatly affected the growth of a-SiNWs. In this work, we adjusted properly the deposition parameters until achieving a-Si:H films with adequate content of hydrogen and with a minimal incorporation of it in form of polyhydrides (Si-H_x_). 

### 2.2. Preparation and Characterization of the Nanowires of Hydrogenated Amorphous Silicon (a-SiNWs)

**Figure 2:**
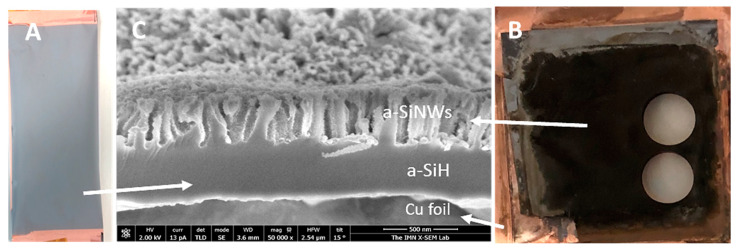
Optical images of a-Si:H films on Cu foil, (**A**) before the MACE process, (**B**) after the MACE process (the appearance of the a-SiNWs to the naked eye is black due to the anti-reflective effect). (**C**) FESEM image, where a-SiNWs formed by MACE, a-Si:H film underlying and bonded to copper that has not been etched, and Cu foil substrate are indicated with labels.

The nanowires were formed by subjecting a-Si:H(i) films deposited directly on a copper foil to a metal-assisted etching process called MACE, as shown in Figure 2a. The Cu foils are 9 microns thick with 99.99% purity (MTI Corporation) and are commonly used as standard metallic collectors in electrodes of LIBs. Before depositing the a-Si:H films, the surface of the Cu foil was cleaned with ethanol to remove impurities that could affect the growth of the material. The grown a-Si:H(i) films were about 1 micron thick and showed excellent adhesion to the rough side of the Cu foil. 

MACE is a two-step etching process that uses Ag as a catalyst. After rinsing the a-Si:H film deposited on Cu foil with deionized water, it is immersed in a first bath consisting of an AgNO_3_/HF aqueous solution (0.025 M/4.8 M). This first step is based on a typical galvanic process, in which Ag^+^ ions of the solution are reduced on the surface of silicon, forming Ag metal aggregates. The silicon beneath these aggregates is, in turn, simultaneously oxidized in the presence of water, producing SiO_2_, which is removed by the HF of the solution. The process proceeds until entire silicon surface is covered by Ag metal nanoparticles. In this work, after carrying out several experiments under different conditions, the optimum time for this first step was stablished at 60 s. Then, samples are dipped in a second bath of an aqueous solution containing H_2_O_2_ as a strong oxidant agent and HF (0.2 M/4.8 M). In this second step, the Ag nanoparticles act as a catalyst promoting the located oxidation and etching of metal-coated silicon, which will ultimately lead to the formation of nanowires. The optimum time of this step will depend on the thickness of the a-Si:H film and its hydrogen content and on the length of nanowires that we want to achieve. Moreover, this time is greatly influenced by the temperature and humidity of the environment. In laboratory working conditions, for this second step, we considered a time range between 45 and 50 s. 

Once the MACE process was finished (Figure 2B), the samples were rinsed with deionized water to remove reagent residues and left to dry in a muffle at 60 °C. The sample surfaces show a dark appearance due to the anti-reflective nature of the nanostructures. 

Finally, field-emission scanning electron microscopy (FESEM), illustrated in Figure 2C, evaluated the lengths, diameters and density of a-SiNW. In addition, we analyzed the chemical composition of the samples both by EDX (energy-dispersive X-ray) to verify that there were no residues from the MACE process in the material before assembling it as an electrode in the battery and by XPS (X-photoelectron spectroscopy) to check that impurities do not appear in the a-Si:H (i) films before MACE etching. According these EDX and XPS measurements, the composition of the samples is mainly silicon (see Supporting Information, Figures S1 and S2).

### 2.3. Assembly and Characterization of the Lithium-Ion Batteries

The assembly of the batteries was carried out in a Jacomex Campus glovebox with an inert atmosphere of argon gas. The amount of H_2_O inside the glovebox is continuously controlled to not exceed 1.0 ppm since the pure lithium to be used, as counter electrode is highly reactive with oxygen. Before introducing them in the glovebox, electrodes were cut into 12 mm diameter circles and left drying overnight inside an oven at 10^−3^ mbar and 90 °C (Büchi B-585) to remove moisture. 

The Li-ion batteries were assembled as CR2032-coin cells in a half-cell configuration. This configuration consists of a working silicon-based electrode and a Li counter electrode placed facing each other and separated by a borosilicate glass fiber separator (Whatman GF/B). The Cu foil on which the a-Si:H film is deposited acts as a positive current collector, and a stain-steel foil placed on top the Li electrode acts as a negative current collector. The electrolyte used was a 1.0 M lithium hexafluorophosphate (LiPF_6_) solution in ethylene carbonate and diethyl carbonate (50/50 (*v/v*)).

For their electrochemical characterization, the batteries were subjected to galvanostatic charge–discharge cycles at 1 Ag^−1^ and at room temperature using an Arbin Instruments BR2143 potentiostat with 12 channels. This method is based on supplying a constant current between the electrodes and measuring the voltage. The capacity (mAh) was determined by multiplying the current by the cycle time. This capacity was normalized to the mass of material, obtaining the so-called specific capacity (mAhg^−1^). The calculation of the specific capacity requires precisely knowing the active material mass. Thus, in the electrodes without nanowires, the active material mass was calculated by weighing the circular Cu collectors with the a-Si:H films deposited on them in a high precision balance (±10^−5^ g) and then subtracting the weight of the collector itself. We repeated this procedure with enough samples so that an average active material mass was estimated. This estimation was in concordance with the calculus made from the density of the a-Si:H film (2.20 ± 0.01 gcm^−3^) and the film thickness obtained from FESEM images. Then, this material density was utilized to determine the active material mass of the circular electrodes once the a-Si:H film thicknesses were measured from SEM images. To obtain the active material mass in nanostructured samples, it is necessary to use a more complicated method, which involves the handling and processing of FESEM images. A detailed description of this method can be found in the reference [15]. 

We evaluated the variation of the specific capacity versus number of cycles for all the materials as anodes studied and compared with the theoretical capacity of graphite to analyze the feasibility of the a-Si:H-based materials as alternative anodes. Galvanostatic charge–discharge (GCD) curves and differential capacity (dC/dV) curves extracted from GCD profiles, for different cycles (1st, 2nd, 10th and 40th), were analyzed in Figure S3 (see Supporting Information).

## 3. Results and Discussion

In previous work [19], hydrogenated amorphous silicon has been recently proven a suitable base material for the synthesis of nanowires by MACE. The etching procedure of this material shows an extraordinary sensibility to the slight compositional changes, such as in the hydrogen content and its bond configuration. We have observed that the way in which hydrogen is incorporated into the amorphous silicon matrix plays a decisive role in whether the etching process successfully produces nanowires. The etching reactivity increases drastically as the bond configuration of hydrogen is shifted from monohydrides (Si-H) to polyhydrides (Si-H_x_). If the proportion of polyhydrides becomes too high, the reactivity will activate a secondary etching process that does not require the catalytic action of the Ag nanoparticles. This secondary etching begins to act on the walls of the formed nanowires shortening them from their tips and could ultimately remove the material from the copper collector. Therefore, the control of the a-Si:H material structure is crucial for this application.

The optical and electrical properties, as well as the composition of the material deposited by PECVD, can be easily adjusted by operating on the different deposition parameters. It has been shown that the substrate temperature is one of the parameters that most influences the hydrogen concentration and its bond arrangement and, as a consequence, the material quality [28]. Thus, an adjustment of the substrate temperature was carried out to achieve a-Si:H films with optimal polyhydrides ratios that are precursors to nanowires with suitable lengths and morphologies. This has been verified through a series of a-Si:H films that were grown with deposition temperatures between 140 °C and 270 °C. The other PECVD deposition parameters for the a-Si:H(i) material were kept fixed: pressure of 53 Pa, radiofrequency density power of 10 Wcm^−2^ and silane flow (SiH_4_, ultrapure 99.999%) of 20 sccm. In Table 1, we show the optical and structural properties of the intrinsic hydrogenated amorphous silicon films tested in this work.

**Figure 3:**
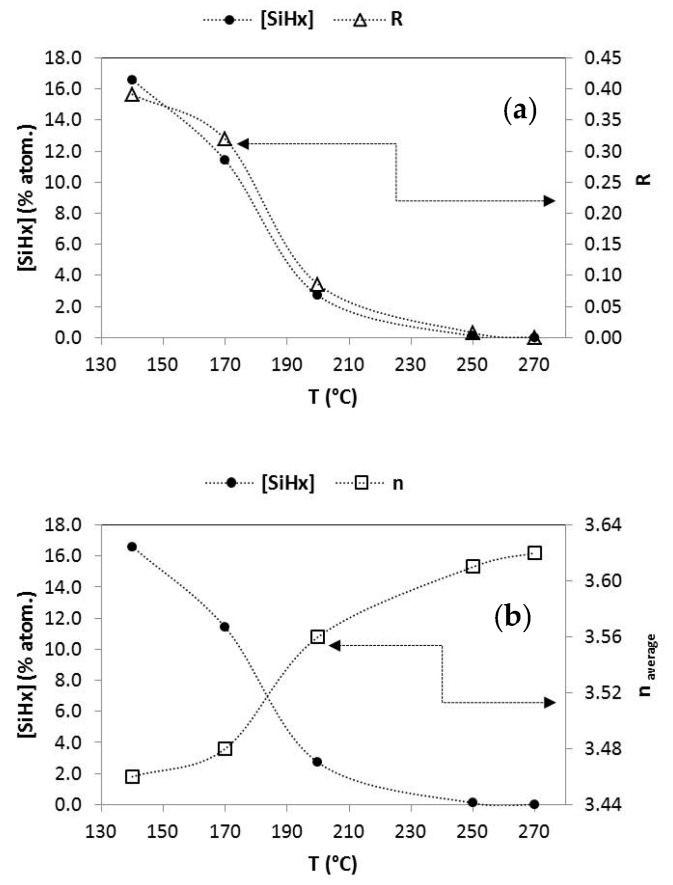
Evolution of the polyhydrides content and (**a**) R factor and (**b**) average refractive index, in the functioning of the deposition temperature of hydrogenated amorphous silicon thin films.

We can clearly observe how the atomic percentage of the hydrogen arranged as polyhydrides ([Si-H_x_]) decreases sharply as the substrate temperature increases in Figure 3. At a temperature around 250 °C, the polyhydrides practically disappear. The term R, previously defined as the quotient between the intensity of the band at 2100 cm^−1^ and the sum of the intensities of the bands at 2100 and 2000 cm^−1^, follows the same trend with temperature (Figure 3a), which indicates that the number of defects and microcavities in the material has been signiflicantly reduced, since the band at 2100 cm^−1^, corresponding to Si-H_x_, is associated with hydrogen atoms attached to the walls of these microcavities. The evolution of the average refractive index (Figure 3b) also reinforces this statement, and the material becomes less porous and with a higher refractive index as the substrate temperature increases. These intrisic a-Si:H films with different hydrogen content were tested to form amorphous silicon nanowires. 

### 3.1. Intrinsic Amorphous Silicon Nanowires

**Figure 4:**
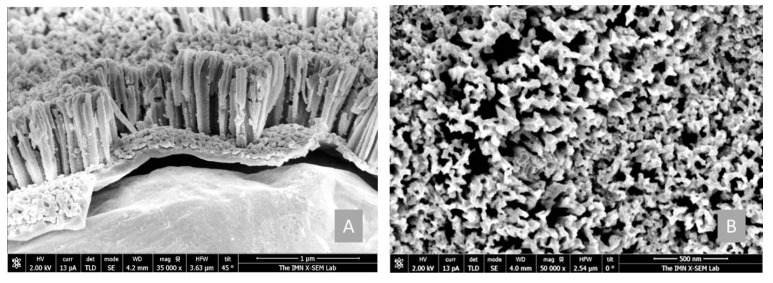
(**A**) Cross section and (**B**) top-view FESEM images of the a-SiNWs obtained after MACE. The a-Si:H(i) base material corresponds to a deposition at 200 ˚C (see Table 1).

Regarding the formation of the nanostructured electrodes, it was performed by subjecting the a-Si:H (i) films of around 1 µm thick deposited on Cu foil to the MACE process described previously. Under these MACE conditions, we achieved uniform and vertical nanowires with lengths of about 850 nm throughout the electrode surface (as can be seen from FESEM images in Figure 4) by using a-Si:H(i) samples with polyhydride content about 3% and total hydrogen content around 14%. On average, the diameter of these nanowires was about 80 nm, according to cross-sectional SEM images. It corresponds to a deposition temperature for a-Si:H(i) films of 200 °C (see Table 1, marked in bold). Similar results were obtained in a previous detailed study [19]. For this reason, a-Si:H(i) deposited at 200 °C and a-SiNWs with the same bulk material were selected as active materials for anodes for the LIBs coin cells analyzed in this work, as will be seen in the next sections. 

### 3.2. n-Doped Hydrogenated Amorphous Silicon 

In order to study how the electrical conductivity of the a-Si:H-based anode affects both specific capacity and capacity retention, electrodes with n-doped a-Si:H films were prepared. To isolate the effect of the conductivity and to be able to compare it with the intrinsic material, it is necessary that the n-doped films have a high conductivity, and their structure is as similar as possible to that of the intrinsic material, regarding the hydrogen content and the polyhydride ratio. However, we have observed the introduction of the phosphine gas (PH_3_) flow rate in the mixture of precursor gases to improve the conductivity of material, inevitably causing a change in the composition of the film, with a greater presence of polyhydrides for high phosphine fluxes. This compositional detail is especially important when doped-a-Si:H layers are deposited.

Therefore, an adjustment of the substrate temperature had to be made so that the material had a minimum amount of hydrogen in the form of polyhydrides (Si-H_x_) without harming its electrical characteristics. The following table, Table 2, shows the properties of doped aSi:H(n) films at different temperatures (200, 236 and 250 °C). The previously optimized undoped a-Si:H(i) at 200 °C was also included in Table 2 for better comparison with n-doped a-Si:H films. 

After incorporating PH_3_, we can see a significant improvement in the electrical properties. An increase in the deposition temperature of a-Si:H(n) films leads to a significant reduction of polyhydrides ([Si-H_x_]) and a decrease in the R factor, while the values of conductivity (σ) and activation energy (E_a_) remain practically unchanged, as does the hydrogen content corresponding to the band at 635 cm^−1^. The doped a-Si:H (n) film compositionally closest to the previously optimized intrinsic a-Si:H(i) was grown at 250 °C (and not at 200 °C as a-Si:H(i)). Still the presence of polyhydrides in this a-Si:H(n) sample was slightly higher than in a-Si:H(i) (4.7 % for n-doped vs. 2.7% for intrinsic a-Si:H films). 

**Figure 5:**
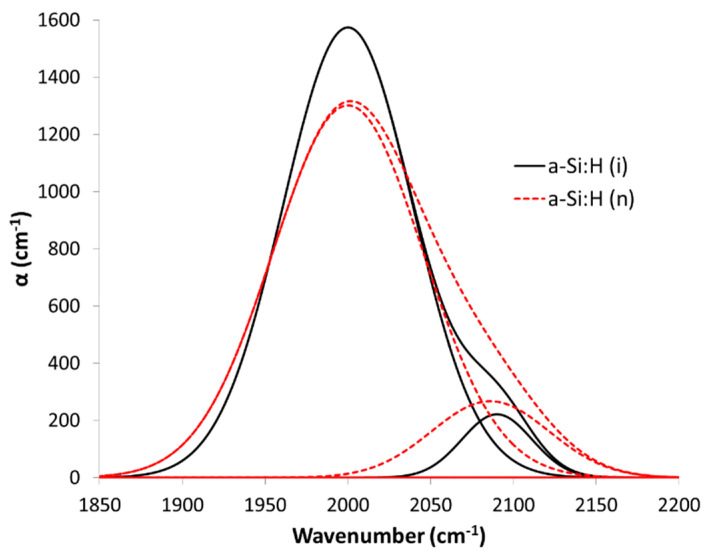
Deconvolution of the FTIR spectra band at 1850–2200 cm^−^^1^ of the a-Si:H(i) film grown at 200 °C and the n-doped a-Si:H film grown at 250 °C, both growing by PECVD, to compare the difference of contribution in the bands centered near 2000 cm^−^^1^ and near 2100 cm^−^^1^.

According to this data on the composition of the hydrogen and as an illustrative example, Figure 5 shows the deconvolution of the FTIR spectra band at 1850–2200 cm^−^^1^ into its two components (one centered near 2000 cm^−^^1^ and the other near 2100 cm ^−^^1^), for the a-Si:H (i) at 200 °C and the a-S:H (n) at 250 °C. We can clearly see a greater contribution at 2100 cm^−^^1^ in the doped material due to a greater presence of polyhydrides (Si-H_x_). The result predicts that the doped films could be a more porous material that would allow it to better withstand volume changes as anode in LIBs and with better electrical properties than the intrinsic material. To check it, both electrodes were proven in LIBs, as we will see in the next section. 

Finally, note that the synthesis of nanowires from these n-doped films was not addressed in this work, since it would require extensive labor to optimize the concentrations of each MACE bath, as well as the etching times, because the presence of phosphorus atoms in the material lattice involves higher polyhydrides ratios and, therefore, a stronger reactivity of the etching. It would be studied in future works.

### 3.3. Electrochemical Performance of the Batteries

The previously optimized a-Si:H(i), a-SiNWs and doped a-Si:H(n) films were employed as anode materials for LIBs to improve their performance.

**Figure 6:**
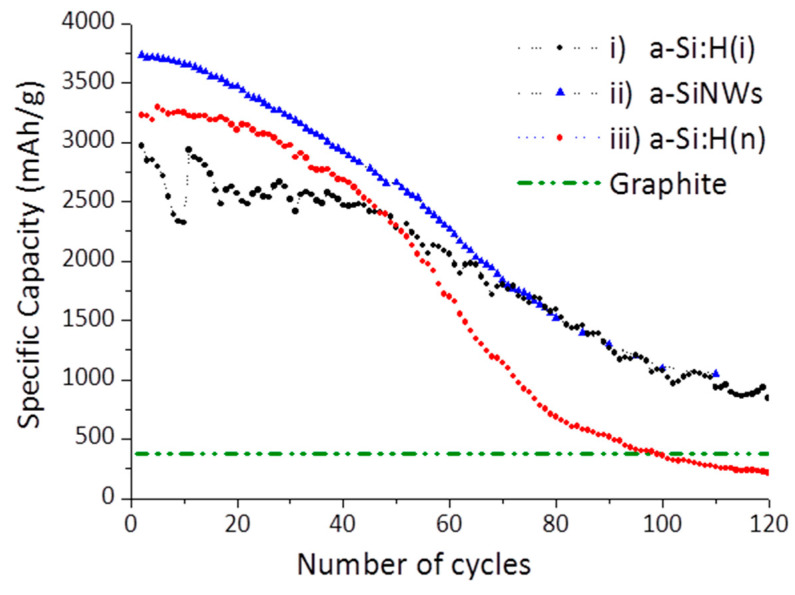
Cycling properties of i)a-Si:H, ii)a-SiNWs and doped iii)a-Si:H(n) at the current density 1 Ag-1. Theoretical graphite density (372 mAhg-1) is included for comparison with the silicon-based materials tested. The initial nonmonotonic behavior of the capacity values of the battery with a-Si:H(i) is due to experimental errors in the potentiostat and not an effect of the material electrode used.

The electrochemical performance of the LIBs was obtained by galvanostatic cycling at 1 A/g. Figure 6 shows the specific capacity versus the number of cycles of the most representative LIBs achieved with these three types of optimized amorphous silicon electrodes. They are compared with the theoretical specific capacity of LIBs with an anode of graphite, that is 372 mAh/g (used in commercial LIBs), wherein we have assumed in this comparison that LIBs with graphite are stable in terms of the number of cycles. We observe that for the first cycles, the specific capacity of the three silicon-based electrodes tested was much higher than the theoretical capacity of the battery with graphite, probably due to the high storage capacity of lithium-ions in the silicon-based materials. This makes hydrogenated amorphous silicon electrodes very promising for commercial LIBs. 

As can be seen in Figure 6, LIBs with non-doped a-Si:H(i) electrodes presented an initial capacity higher than 2500 mAhg^−^^1^ and remained fairly stable with the number of cycles, with a capacity retention greater than 80% after fifty charging cycles. This initial capacity has been increased remarkably when LIBs were assembled with nanostructured silicon electrodes, with respect to the bulk a-Si:H (i). This difference in the specific capacity could be due to less stress in a-SiNWs during the volumetric expansion, since the nanostructured materials present a higher surface/volume ratio that increases the surface in contact with the electrolyte. Furthermore, nanostructured materials, such as the a-SiNWs, could reduce the diffusion length of Li-ions to improve the performance of the battery. This initial capacity in a-SiNW-LiBs (3800 mAhg^−^^1^) was nearly 10 times higher than the theoretical capacity of graphite and very close to the maximum theoretical capacity for silicon-based electrodes (4200 mAhg^−^^1^) [36], so a priori it can be considered an excellent result, the first result with this type of nanostructured amorphous anode. Unfortunately, both LIBs gradually decay with the cycle number, and after very long cycling, the specific capacity of the a-SiNW-LIBs is very similar that of a-Si:H(i)-LIBs, which could be produced by the deterioration of the nanostructures due to the volume changes in the charge and discharge processes. Despite the decrease in its capacity with the number of cycles, the specific capacity for both electrodes continues to be much higher than graphite after hundreds of cycles. 

Regarding the third type of LIBs tested, doped electrodes of a-Si:H(n) films, without nanostructures, were also proven. A high initial capacity was obtained (3250 mAhg^−^^1^), versus intrinsic a-Si:H (2500 mAhg^−^^1^), probably due to the upper conductivity of a-Si:H(n) films. It was attributed to the improvement of the electrical conductivity of doped amorphous silicon films and to a reduction of the resistance to the charge transfer [9]. On the contrary, the specific capacity decreased faster than the other two types of non-doped electrodes tested, especially after fifty cycles (marked in the figure) and falling below the theoretical value of graphite after 100 cycles. This effect does not occur in the other intrinsic silicon materials assayed. An explication of this falling could be the unavoidable larger presence of polihydrides (SiH_x_) in the doped material (see [Si-H_x_] and R-factor in Table 2), as a consequence of the incorporation of PH_3_ diluted to 2% in H_2_ in the mixture of gases (that is, together with SiH_4_) during the growing of a-Si:H(n) by PECVD. This [Si-H_x_] content is generally associated with the presence of micropores and microstructures that may be anomalous in the material. Although, initially, we considered that the presence of a greater quantity of polihydrides in these conductive layers could be favorable to support the tensions derived from the changes in volume and help in the diffusion of lithium in the anode; on the contrary, we have observed experimentally in the LIBs cycling a faster deterioration. This doped material is likely to fracture more rapidly due to the higher concentration of SiH_x_, and therefore the battery life will be shorter. Therefore, we consider that a balance is necessary between the level of doping (which increases conductivity and improves initial capacity) and the presence of polyhydrides (which can cause a loss of longevity). Both properties can be controlled and modified by acting on the PECVD deposition parameters. It is a great advantage of this growing technique for this type of electrodes that there is a possibility of tuning the properties of the deposited material. 

In our laboratories, a more exhaustive study is beginning to be carried out on a-Si:H(n), which will allow us to analyze the behavior of LIBs based on the concentration of hydrogen and its distribution and with respect to its doping level. After optimization, it is very likely that LIBs could obtain thin-film n-doped amorphous silicon electrodes with capacities very close to those achieved with a-SiNWs and in a much simpler way, since it would not be necessary to etch them chemically with the MACE method.

## 4. Conclusions

Electrodes based on thin films of hydrogenated amorphous silicon (intrinsic and doped) growing by PECVD and amorphous silicon nanowires have been analyzed as alternative materials to conventional graphite electrodes for lithium-ion batteries. It has been observed how the PECVD deposition temperature of the amorphous silicon films and the incorporation of dopants notably influence the compositional structure of the material and their conductivity. The nanostructuring of silicon thin-films using MACE etching processes has been optimized to obtain undoped amorphous silicon nanowires, directly adhered to the copper collector. It is a huge advantage over crystalline silicon nanowires grown by other methods, as they would need a binder to stick to the copper film. 

All three types of amorphous silicon electrodes have been tested on LIBs. The electrochemical results obtained have been very promising, with specific capacities far superior to the theoretical capacity of graphite. The best result has been obtained with amorphous silicon nanowires, with an initial capacity of 3800 mAhg^−1^, probably due to an increase in the specific surface that allows a greater storage of Li-ions. On the other hand, after a large number of cycles, its capacity decreases and equals that of electrodes based on intrinsic amorphous silicon in thin films, but both remain well above that of graphite. In relation to the electrodes with doped amorphous silicon, we also obtained a high initial capacity, greater than 3000 mAhg^−1^. In contrast, the stability and longevity of these batteries were markedly lower than batteries with electrodes of intrinsic material. This result has led us to suspect that the greater inherent presence of polyhydrides in the doped material causes an accelerated deterioration in the batteries. Since the hydrogen content and the doping level in the doped amorphous silicon layers are perfectly controllable by the deposition parameters of the PECVD technique, it will be of great interest to reach a balance between both parameters. The authors are already analyzing in detail the influence of the presence of polyhydrides in the lithiation processes and in the stability of the batteries for imminent future research. 

## Figures and Tables

**Table 1:** Optical and structural properties of a-Si:H(i) films deposited by PECVD with different hydrogen composition.

T (°C)	n_average_ (300–2500 nm)	[H_635_] (%)	[Si-H] (%)	[Si-H_x_] (%)	R
140	3.46	21.5	11.2	16.6	0.39
170	3.48	19.6	10.1	11.4	0.32
**200**	**3.56**	**14.1**	**11.0**	**2.7**	**0.09**
250	3.61	10.2	8.2	0.1	0.01
270	3.62	10.9	8.9	-	-

**Table 2:** Optical, electrical and structural properties of a-Si:H(n) and a-Si:H(i) films deposited by PECVD.

a-Si:H	T (°C)	n	[H_635_] (%)	[Si-H] (%)	[Si-H_x_] (%)	R	σ (S/cm)	E_a_ (eV)
i	200	3.56	14.1	11.0	2.7	0.09	4.4 × 10^−10^	0.84
n-doped	200	3.46	14.8	8.5	8.4	0.29	1.4 × 10^−2^	0.18
	236	3.53	13.8	10.8	5.6	0.17	2.0 × 10^−2^	0.19
	250	3.56	13.7	11.7	4.7	0.13	1.5 × 10^−2^	0.18

## Data Availability

The data is available on reasonable request from the corresponding author.

## Supplementary Materials

The following supporting information can be downloaded at: https://www.mdpi.com/article/10.3390/nano12244400/s1, Figure S1: EDX (Energy-Dispersive X-ray) measurements of amorphous silicon nanowires grown on Cu foil. The composition of these samples is fundamentally silicon. The presence of silver (Ag) is due to the silver nanoparticles present as a catalyst, necessary for the MACE etching to form the aSiNWs. The presence of copper is due to Cu-foil as collector of current; Figure S2: XPS (X-Photoelectron Spectroscopy) measurements of intrinsic amorphous silicon thin-film before MACE etching. The composition of these samples is fundamentally silicon, and not impurities appear; Figure S3: Galvanostatic Charge-Discharge (GCD) curves (left column) and Differential Capacity (dC/dV) curves (right column) extracted from GCD profiles, for different cycles (1st, 2nd, 10th and 40th) as labeled of the LIBS with intrinsic a-Si:H thin films and a-Si:H nanowires (a-SiNWs) as electrodes.
