# Supplementary material for: Hydrogenated Amorphous Silicon-Based Nanomaterials as Alternative Electrodes to Graphite for Lithium-Ion Batteries
Source: Nanomaterials (Basel). 2022 Dec 9;12(24):4400. doi: 10.3390/nano12244400 (PMC9785924; doi:10.3390/nano12244400)
Supplement: Supplementary file 1 [file nanomaterials-12-04400-s001.zip › nanomaterials-2058998-supplementary.pdf]

nanomaterials

Supplementary Materials:

EDX (Energy-Dispersive X-ray) measurement

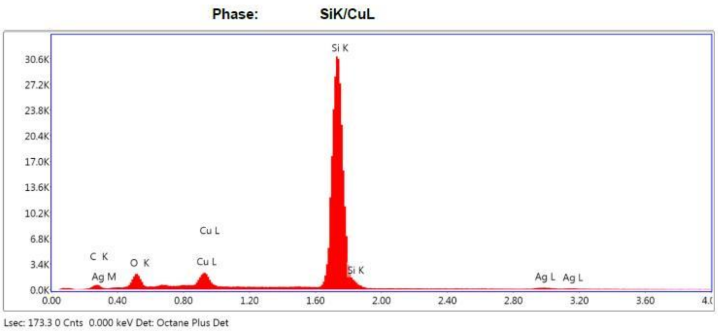

eZAF Smart Quant Results

Element	Weight %	Atomic %	Error %
C K	3.36	7.74	12.61
O K	3.55	6.13	7.82
CuL	4.57	1.99	5.26
SiK	84.41	83.09	4.41
AgL	4.12	1.06	22.12

Figure S1. EDX (Energy-Dispersive X-ray) measurements of amorphous silicon nanowires grown on Cu foil. The composition of these samples is fundamentally silicon. The presence of silver (Ag) is due to the silver nanoparticles present as a catalyst, necessary for the MACE etching to form the aSiNWs. The presence of copper is due to Cu-foil as collector of current.

XPS (X-Photoelectron Spectroscopy) measurement

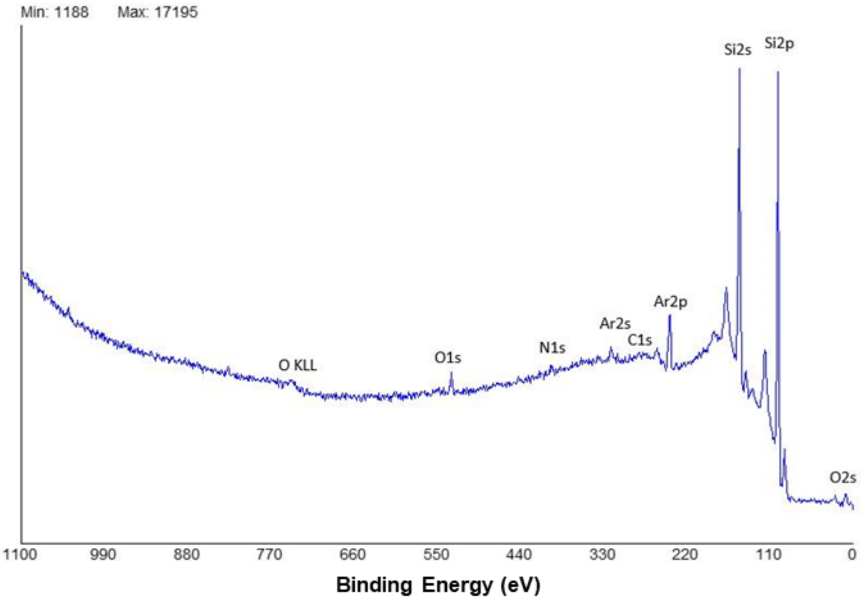

Figure S2. XPS (X-Photoelectron Spectroscopy) measurements of intrinsic amorphous silicon thin-film before MACE etching. The composition of these samples is fundamentally silicon, and not impurities appear.

Galvanostatic Charge-Discharge (GCD) and Differential Capacity (dC/dV) for different cycles (1st, 2nd, 10th and 40th) of the LIBs

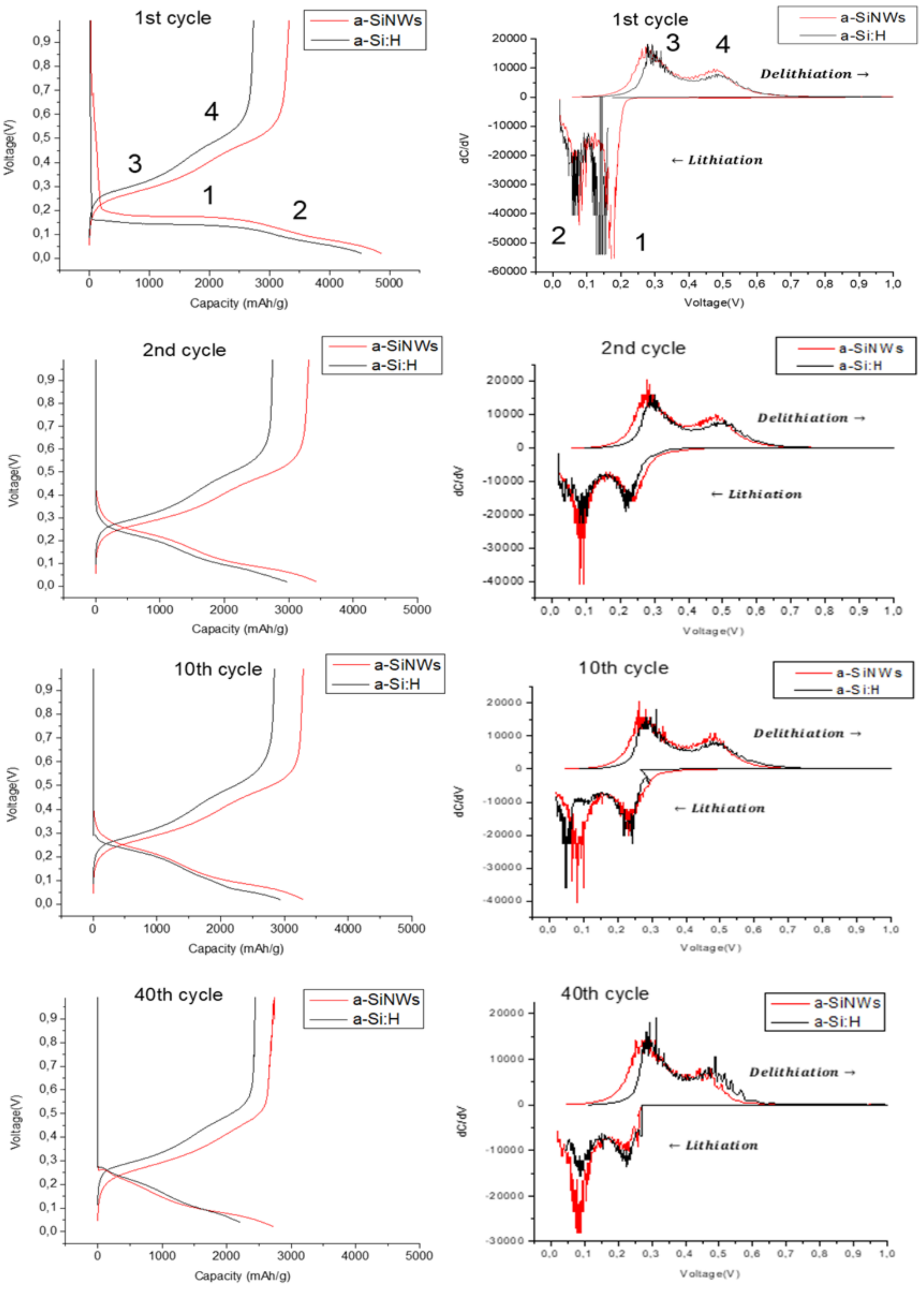

Figure S3. Galvanostatic Charge-Discharge (GCD) curves (left column) and Differential Capacity (dC/dV) curves (right column) extracted from GCD profiles, for different cycles (1st, 2nd, 10th and 40th) as labeled of the LIBS with intrinsic a-Si:H thin films and a-Si:H nanowires (a-SiNWs) as electrodes.

Supplementary explanation Figure S3

The Galvanostatic Charge-Discharge (GCD) curves for different cycles are shown in Figure S3 (left side). From these profiles, the differential capacity (dC/dV) curves were extracted (right side).

In all the differential curves of the figure (right column) there are two relatively broad peaks (peaks 1 and 2) corresponding to the lithiation processes, indicating one-phase transition reactions, since Si is amorphous and transforms into another phase too. On the contrary, if we had used crystalline silicon, the peaks in the curve associated to the 1st cycle should appear very narrow (a cathodic peak ~0.15 V) associated with irreversible amorphization processes from crystalline Si to amorphous Li_xSi phase, a two-phase transition.

The exact compounds formed during the lithiation cycle are not known, but it is assumed that there are two consecutive incorporations of Li to amorphous Si-Li phases, following the next reactions:

$a\text{-}Si + xLi \leftrightarrow a\text{-}Li_xSi$

$a\text{-}Li_xSi + yLi \leftrightarrow a\text{-}Li_{x+y}Si$

The peaks 3 and 4 correspond to the inverse reactions in the delithiation process.

In the dC/dV curves, the intensity of the lithiation peaks in the 1st cycle is significantly greater than in the 2nd and subsequent cycles, possibly caused by irreversible reactions with the electrolyte leading to the formation of the SEI layer (Solid Electrolyte Interface). After 2nd cycle, the intensity of the peaks decreases due to capacity loss with the cycling. The higher peak intensities of the a-SiNWs (red) in all represented cycles reveal a larger amount of material activated during lithiation/delithiation with this nanostructuring.
